# 172. Impact of 24/7 Rapid Diagnostic Testing Pharmacist Interventions and Infectious Diseases Consultation in Pediatric *Staphylococcus aureus* Bacteremia

**DOI:** 10.1093/ofid/ofae631.052

**Published:** 2025-01-29

**Authors:** Catherine Ann C Aquino, Paul Gavaza, Maulin Soneji, Noreen Chan Tompkins

**Affiliations:** Loma Linda University School of Pharmacy, Sun Valley, CA; Loma Linda University School of Pharmacy, Sun Valley, CA; Children's Hospital of Philadelphia, Philadelphia, Pennsylvania; Loma Linda University Children's Hospital & School of Pharmacy, Loma Linda, California

## Abstract

**Background:**

The benefits of pharmacist interventions using rapid diagnostic testing (RDT) and Infectious Diseases (ID) consultation for *Staphylococcus aureus* bacteremia (SAB) have been documented in adults, but data on pediatric patients is sparse. The study objectives were to evaluate the impact of 24/7 pharmacist-led RDT interventions and pediatric ID consultation on clinical outcomes in pediatric SAB.

**Figure:**
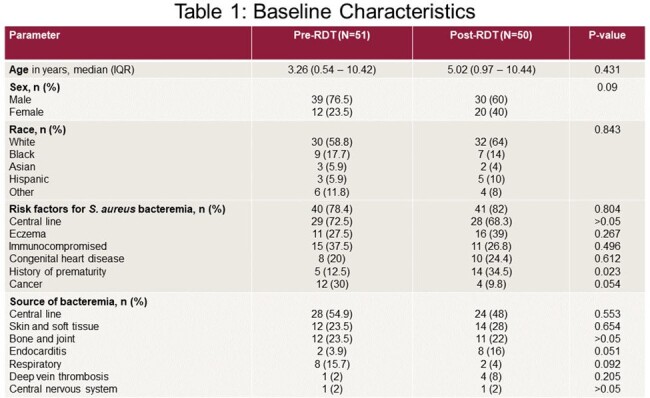

**Methods:**

This retrospective cohort study analyzed pediatric patients (age < 18 years) with SAB admitted to Children’s Hospital comparing outcomes before and after implementation of 24/7 pharmacist-led RDT interventions. Pre-intervention (Pre-RDT) group included patients admitted from February 2013 to January 2015 and the post-intervention (Post-RDT) group from August 2021 to July 2023. Premature infants (< 36 weeks gestational age) and patients with polymicrobial bacteremia or fungemia were excluded.

**Figure:**
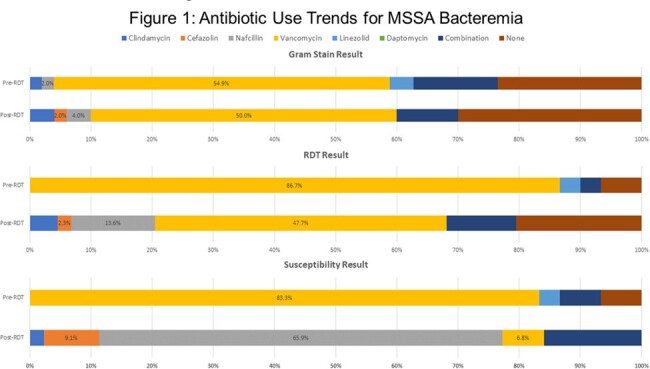

**Results:**

A total of 101 patients were included: Pre-RDT (n=51) and Post-RDT (n=50). Most patients were male (68.3%) and white (61.4%) with similar baseline characteristics (Table 1). The median time to optimal antibiotic (TOAT) from blood culture collection was lower in the Post-RDT group than the Pre-RDT group (23.9 vs 29.0 hours; p=0.673) for MRSA and MSSA isolates. The Post-RDT group had lower median TOAT for MSSA (24.8 vs 87.7 hours; p < 0.001) with higher use of beta-lactams (Figure 1), lower hospital length of stay (LOS) (12 vs 17 days; p=0.007), lower ICU admissions (40.0% vs 64.7%; p=0.017), and shorter ICU LOS (14 vs 17 days; p=0.022) than patients in the Pre-RDT group. Post-RDT patients had fewer 30-day readmission (22.0% vs 23.5%; p > 0.05) and in-hospital mortality (4% vs. 7.8%; p > 0.05) than Pre-RDT patients. ID was consulted more frequently in the Post-RDT group (94.0% vs. 56.9%; p < 0.001). In-hospital mortality was lower with ID consultation than without it (2.6% vs. 16.0%; p=0.032).

**Conclusion:**

Integration of 24/7 RDT pharmacist interventions and pediatric ID consultations were associated with improved clinical outcomes (TOAT for MSSA, hospital and ICU LOS and in-hospital mortality) in pediatric patients with SAB, highlighting the effectiveness of these interventions in a pediatric antimicrobial stewardship framework.

**Disclosures:**

**All Authors**: No reported disclosures

